# Presence of one ecto- and two endoparasite species of the black stork (*Ciconia nigra*) in Portugal

**DOI:** 10.1186/s12917-020-02724-6

**Published:** 2021-01-07

**Authors:** David W. Ramilo, Inês Caetano, Erica Brazio, Manuela Mira, Leonor Antunes, Isabel Pereira da Fonseca, Luís Cardoso

**Affiliations:** 1 CIISA – Centro de Investigação Interdisciplinar em Sanidade Animal, Faculdade de Medicina Veterinária, Universidade de Lisboa, Avenida da Universidade Técnica, 1300-477 Lisboa, Portugal; 2 LxCRAS – Wild Animal Rehabilitation Centre of Lisbon, Lisbon, Portugal; 3 Student of the Integrated Master in Veterinary Medicine, Faculdade de Medicina Veterinária, Universidade de Lisboa, Avenida da Universidade Técnica, 1300-477 Lisboa, Portugal; 4 Department of Veterinary Sciences, and Animal and Veterinary Research Centre (CECAV), University of Trás-os-Montes e Alto Douro (UTAD), Vila Real, Portugal

**Keywords:** Black stork, *Cathaemasia*, *Desportesius*, *Neophilopterus*, Portugal

## Abstract

**Background:**

The black stork (*Ciconia nigra* Linnaeus, 1758) is a recognized endangered species in Europe and most of the specimens from the Western Palearctic region breed in the Iberian Peninsula. Available works regarding parasites in black storks are scarce. This work reports the presence one ecto- and two endoparasite species from a black stork in Portugal.

**Case presentation:**

A black stork was found in southern Portugal after colliding against electric cables. The specimen did not survive its sustained injuries and a post-mortem exam was performed. During the procedure, several ecto- and endoparasite specimens were found. The collected parasites were lice (*Neophilopterus tricolor*), nematodes (*Desportesius sagittatus*) and trematodes (*Cathaemasia hians*).

**Conclusions:**

Three different species of parasites are reported from a black stork in Portugal. Ecto- and endoparasites of *C. nigra* have not frequently been described in the literature, and this case report is a contribution to the field. Additional studies will be important to better understand the impact that parasites can have on *C. nigra* health and survival.

## Background

The black stork (*Ciconia nigra* Linnaeus, 1758) is a threatened species internationally protected and listed in Annex I of the EU Birds Directive [1]. This long-distance migratory bird has a wide territorial distribution, with more than 50% of their European population distributed across Eastern Europe [2-4]. On the other hand, this species is very rare in Western Europe, where it has suffered a drastic reduction due to the destruction of its natural habitat [5]. Among the black stork reproduction areas in Europe, western Spain, bordering Portugal, can be mentioned [5]. In mainland Portugal, this bird occurs inland, mainly along the hydrographic basins of Tagus, Douro and Guadiana rivers [6]. There are about 100 nesting couples in Portugal and some of these specimens are resident during the winter [6, 7]. Usually, black storks migrate to Africa in the autumn, returning to Europe during the spring [8, 9].

Information concerning parasitological fauna found in *C. nigra* is available in a few published works, but data are still scarce and more studies are necessary [9–14]. The main reason for this lack of information is related to the black stork’s habitat, since these birds breed in dense wood areas where precise nesting surveys are difficult to carry out. Moreover, because they are included in the list of protected animals in Europe [9], human contact should be avoided in order to not disturb them in their natural habitat.

This report describes three different parasite species found in a black stork from Portugal.

## Case presentation

An adult female black stork was found near Alqueva dam (38°11’51’’N, 7°29’47’’W), within the boundaries of the districts of Beja and Évora, southern Portugal, in March 2018, after colliding against electric cables. The specimen was received at the Wild Animal Rehabilitation Centre of Lisbon (LxCRAS) with several wounds, including an extensive hematoma in the pectoralis muscles, in the cranial zone of the keel and in the triceps muscle. After 3 days of treatment, the bird died due to the injuries and the corpse was sent to the Pathology Service of the Faculty of Veterinary Medicine of the University of Lisbon for *post-mortem* examination. During the procedure, trematodes that were present in the bird’s mouth showed photophobic behaviour, moving back to the oesophagus when the beak was opened. Ectoparasites were collected in 70% ethanol and later placed on slides with lactophenol or Canada balsam [15] and observed under light microscopy. Collected endoparasites were placed in a Petri dish containing saline solution before microscopic examination.

Adult stages of three different parasites were found: lice on breast and belly feathers (*n* = 26), nematodes in the gizzard (*n* = 2) and trematodes in the oesophagus (*n* = 27). For parasite species’ identification, several references were used [9, 10, 12, 16]. Parasites measurements can be observed in Tables 1, 2 and 3.

**Table 1:** Measurements of male and female lice collected from *Ciconia nigra*

Measurement (mm)	Males (*n* = 10)			Females (*n* = 7)		
	Minimum	Maximum	Average	Minimum	Maximum	Average
Cephalic length	0.57	0.67	0.61	0.68	0.77	0.74
Cephalic width	0.74	0.83	0.79	0.82	0.94	0.90
Thoracic length	0.39	0.46	0.43	0.45	0.52	0.48
Thoracic width	0.54	0.69	0.62	0.72	0.84	0.78
Abdominal length	1.15	1.39	1.30	1.62	2.13	1.91
Abdominal width	0.78	1.08	0.96	0.93	1.28	1.20
Total length	2.11	2.52	2.34	2.75	3.42	3.13

**Table 2:** Measurements of female nematodes collected from *Ciconia nigra*

Measurement (mm)	Females (*n* = 2)
Length	6.12–6.36
Width	0.219–0.252
Distance of vulva to the tip of the tail	0.083
Eggs (length x width)	0.03 × 0.017

**Table 3:** Measurements of trematodes collected from *Ciconia nigra*

Measurement (mm)	Specimens (*n* = 10)		
	Minimum	Maximum	Average
Length	5.76	7.20	6.50
Width	2.78	3.86	3.24
Ventral sucker diameter	9.16	15.48	11.84
Oral sucker diameter	6.84	8.95	8.09

**Fig. 1:**
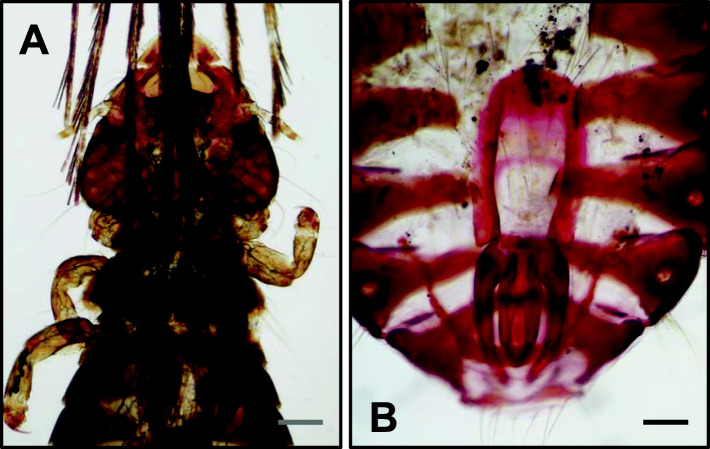
*Neophilopterus tricolor*. **a**: head of female specimen (scale bar: 231.4 µm); **b**: abdomen of male specimen (scale bar: 210.8 µm)

After preparation of lice by the Canada balsam technique, they were identified as *Neophilopterus tricolor* (Burmeister, 1838) (Fig. 1a and b), due to the presence of a fifth shorter marginal temporal seta when compared with the size of the other four setae. The performed measurements (Table 1) were in agreement with those registered for this species [10].

**Fig. 2:**
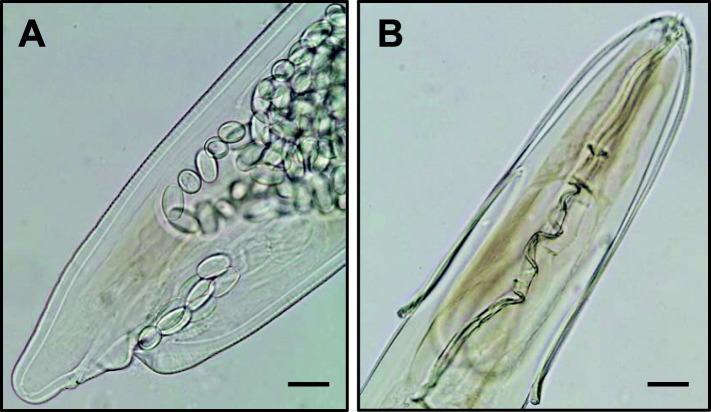
*Desportesius sagittatus* female specimen. **a**: posterior end (scale bar: 34 µm); **b**: anterior end (scale bar: 67.4 µm)

Following endoparasite observation, nematodes were recognized as *Desportesius sagittatus* (Rudolphi, 1809) (Fig. 2a and b). *Desportesius sagittatus* can be identified by length and morphology of the male right spicule and the greatly reduced deirids [16]. Since only two female specimens were found, the identification was made by this species’ type host (i.e. *C. nigra*), the presence of reduced deirids and the performed measurements (Table 2), which matched those previously observed for this species [16, 17].

**Fig. 3:**
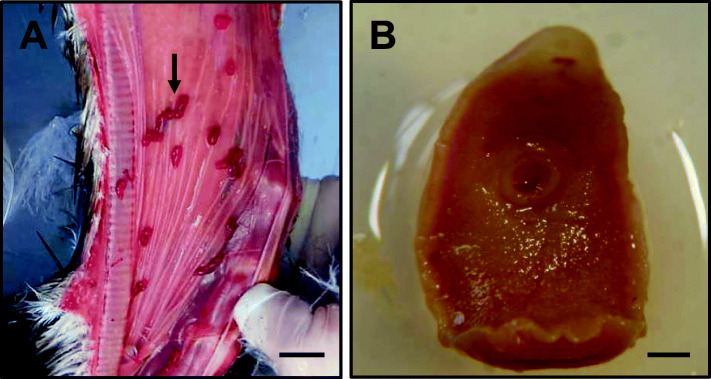
*Cathaemasia hians*. **a**: Specimens (black arrow) on oesophagus (scale bar: 14.4 mm); **b**: specimen ventral view (scale bar: 910 µm)

Trematodes were identified as *Cathaemasia hians* (Rudolphi, 1809) (Fig. 3a and b). The observed specimens had flattened body, presence of scales on the ventral cuticle and the head crown was lacking (Cathaemasiidae family). The obtained measurements were in agreement with those observed for this species (Table 3). Eggs (*n* = 5) had a mean length of 98.3 µm and an average width of 52.8 µm. These findings, together with the host and their localization, allowed species identification [9, 12, 13, 18, 19].

## Discussion and conclusions

In the present work, three different parasites of *C. nigra* were observed during necropsy of an adult specimen. The genus *Neophilopterus* (Ischnocera: Philopteridae) has already been described in black storks from Spain [10]. Ischnocera lice can affect host thermoregulation and induce feather breakage, reducing host fitness through the energetic consequences of that damage. Stress is also an indirect effect to the affected host [10]. Lice specimens found in this report’s black stork had four marginal temporal setae of approximately the same length but the last one was several times shorter, thus being identified as *Neophilopterus tricolor*. This is the first reference of this species in a black stork in Portugal.

In what refers to nematodes, until the year 2015, only Austria, Czech Republic, Germany, Poland and Slovakia had information regarding helminth communities in storks [13]. The genus *Desportesius* (Nematoda: Acuarioidea) usually parasitizes birds of the order Ciconiiformes [17]. These nematodes occur under the gizzard lining [16], but nothing is known about their pathogenic effects on the hosts [14]. *Desportesius sagittatus* has also been reported in *C. nigra* [13]. However, this is the first reference of this species in a black stork caught in mainland Portugal.

Considering trematodes, *Cathaemasia hians* (Trematoda: Cathaemasiidae) is a well-known species which has members of family Ciconiidae as definitive hosts and are generally found in the oral cavity and sometimes in the oesophagus of these birds. However, reports of this species in Europe have been rare [12]. Black storks are more likely to be infected with this parasite than white storks (*Ciconia ciconia*), due to their different feeding behaviour [18]. In fact, white storks feed on arthropods and earthworms from permanent dry pastures [20, 21], and black storks ingest amphibians and fish parasitized with metacercariae of *Cathaemasia hians*, which live in swamps and slow-flowing waters [12,18]. *Cathaemasia hians* is generally considered as non-pathogenic to storks [12, 13]. Nevertheless, this parasite can lead to irreversible alterations in the digestive tract of definitive hosts, lowering the birds’ fitness. Massive infections by this parasite can cause serious health problems when combined with cachexia or lower immunity of the hosts [9, 13]. This is also the first reference for this parasite in a black stork captured in Portugal.

Scientific studies regarding these birds are difficult to accomplish since they are hard to access and protected by European laws. Thus, living animals can only be found after sustaining serious injuries [9, 12, 18], like in the present study.

Since parasitological works in *C. nigra* are scarce, this work is considered as an important contribution to this study field, reporting three parasite species in a black stork caught in mainland Portugal.

## Data Availability

The datasets used and/or analysed during the current study are available from the corresponding author on reasonable request.

## Abbreviations

*C.*: *Ciconia*
EU: European Union
i.e.: *id est*, that is
LxCRAS: Wild Animal Rehabilitation Centre of Lisbon
mm: Milimeter
N: North
n: Sample size
W: West
µm: Micrometer
